# Arsenic Exposure and Breast Cancer Risk: A Re-Evaluation of the Literature

**DOI:** 10.3390/nu12113305

**Published:** 2020-10-28

**Authors:** Katherine Pullella, Joanne Kotsopoulos

**Affiliations:** 1 Department of Nutritional Sciences, University of Toronto, Toronto, ON M5S 1A1, Canada; katherine.pullella@wchospital.ca; 2 Women’s College Research Institute, Women’s College Hospital, Toronto, ON M5S 1B2, Canada; 3 Dalla Lana School of Public Health, University of Toronto, Toronto, ON M5T 3M7, Canada

**Keywords:** arsenic, breast cancer, environmental toxin, carcinogen, human exposure, review

## Abstract

Arsenic is a widespread environmental contaminant and recognized carcinogen for the skin, bladder and lungs. In recent years, there has been an increasing number of studies that have investigated the effects of arsenic exposure and cancer risk at other sites, including the breast. However, to date, the association between arsenic exposure and breast cancer risk remains unclear. This article will provide an overview of arsenic metabolism, the clinically important biomarkers commonly used to assess arsenic exposure, and review the epidemiologic studies examining the role of arsenic exposure on breast cancer risk. Given the large burden of disease associated with breast cancer, it is of the upmost importance to identify risk factors and preventative strategies that could reduce cancer incidence. Limiting exposure to endemic environmental toxins, such as arsenic, represents one such strategy. More studies are required to better ascertain this relationship and to develop the public policy necessary to significantly reduce breast cancer incidence.

## 1. Introduction to Arsenic

Arsenic, a widespread environmental contaminant, is a highly toxic metalloid that represents a prominent component of the earth’s crust [1]. Through both natural, and anthropogenic events, arsenic has become omnipresent in the natural world; tainting soil, rock beds, water supplies and air [2,3,4]. The primary route of human exposure to arsenic is through the consumption of contaminated water or food (e.g., seafood, fish or rice) as well as from smoking tobacco [1,5]. Various occupations and hobbies, including mining, non-ferrous smelting, electronic manufacturing, pesticide production and woodworking, may also increase the risk of the inhalation of arsenic species, and are less common sources of exposure [5,6,7]. Exposure to any amount of arsenic is of concern as arsenic has been linked to several adverse health outcomes including impaired neurological development, cardiovascular disease, diabetes, and cancer incidence [6]. In 1993, the World Health Organization (WHO) established the provisional limit of arsenic in drinking water to be under 10 ug/L [8,9]. Recent estimates suggest that over 200 million people have been exposed to elevated levels of arsenic from drinking water alone, underscoring the fact that arsenic contamination continues to be a significant public health concern on a global scale [6].

Arsenic compounds exist in one of three groups, each with a differing level of toxicity: (1) inorganic arsenic compounds (iAs), (2) organic arsenic compounds (oAs) and (3) arsine gas [5,10,11]. Inorganic arsenic and arsine gas are recognized as highly toxic; while organic arsenic is deemed relatively non-toxic, though toxicity can accumulate if found in high enough concentrations [12,13]. Inorganic arsenic is recognized as a *bona fide* carcinogen by the International Agency for Research on Cancer (IARC) for cancers of the skin, bladder and lung [5]. To date, the vast majority of literature examining the association between arsenic exposure and cancer risk has exclusively focused on these sites; however, more recently there has been an increasing number of studies investigating the impact of arsenic exposure and cancer risk at other sites, including the breast.

Breast cancer is the most commonly diagnosed cancer in women worldwide, with an increasing number of new cases each year [14,15]. Given the large burden of disease associated with breast cancer, it is of the upmost importance to identify risk factors and preventative strategies that could reduce cancer incidence [15]. Epidemiologic studies have revealed the important role of family history and genetic predisposition, as well as various hormonal and reproductive exposures that may increase breast cancer risk. The role of lifestyle and dietary factors have also been explored but preventative strategies remain limited. Reducing exposure to environmental toxins through dietary regulation and public health policy represents one potential preventative strategy that has, to date, been understudied. Of interest is the potential impact of trace elements, including arsenic status, and the risk of breast cancer. This review will provide an in-depth examination of the current state of the literature on the relationship between arsenic and breast cancer by reviewing arsenic metabolism, the clinically important biomarkers used to assess arsenic exposure, and the epidemiological studies investigating arsenic exposure and subsequent breast cancer risk.

## 2. Overview of Arsenic Metabolism and Epigenetic Modifications

**Figure 1:**
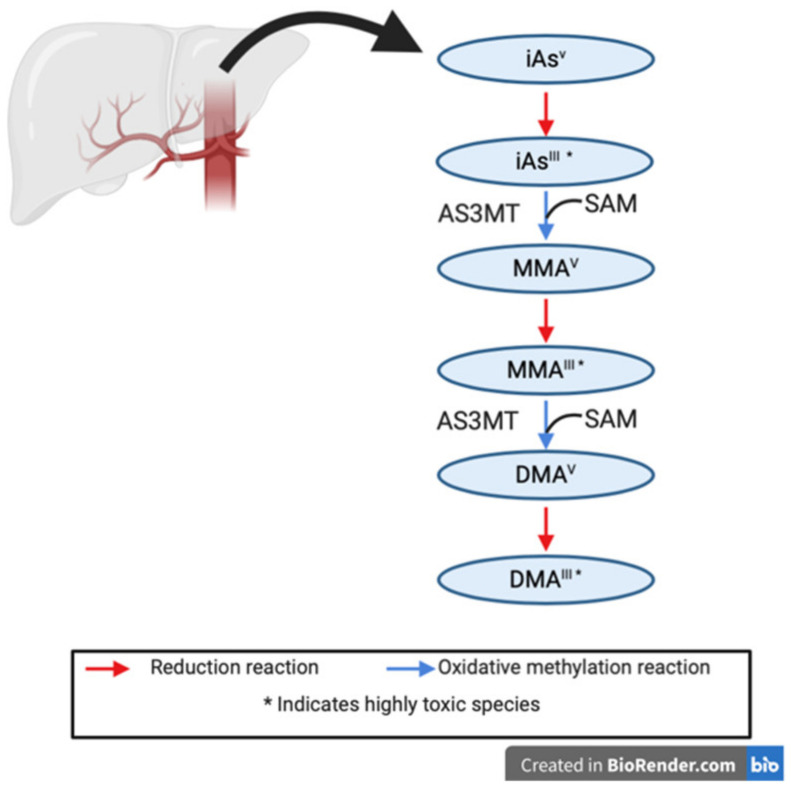
Inorganic arsenic metabolism pathway as proposed by Challenger, 1947. Inorganic arsenic metabolism occurs in the liver, by a series of oxidative methylation and reduction steps. Methylation occurs using the arsenic-3-methyltransferase (AS3MT) enzyme, and methyl donor S-adenosylmethionine (SAM).

Arsenic compounds can be ingested from food and water sources, absorbed by the skin from the surrounding environment and water supplies (e.g., showering, swimming, and pesticides), and inhaled from the air. Since inorganic arsenic is the only IARC identified carcinogen, previous studies characterizing arsenic metabolism and detoxification have primarily focused on this compound. Studies investigating inorganic arsenic metabolism have been ongoing since the 19th century; yet, the exact detoxification pathway for this toxin still lacks clarity within the literature [10,16]. In 1947, Frederick Challenger was the first to propose a reductive-oxidative methylation pathway for the detoxification of inorganic arsenic into methylated species that are easily excreted in the urine [17]. This pathway, from inhalation or ingestion to urination, forms the backbone of arsenic metabolism, and is summarized in Figure 1 [16,18]. Inorganic arsenic ingested from contaminated food and water sources, and environmental sources, is commonly found in the highly toxic trivalent (iAs^III^) or pentavalent state (iAs^V^) [5,18,19]. Challenger proposed that arsenic-3-methyltransferase (AS3MT), coupled with S-adenosylmethionine (SAM), could methylate ingested trivalent arsenite to the first intermediate, monomethylarsonic acid (MMA^V^), in the hepatocytes of the liver [20,21]. This intermediate is then further reduced to monomethylarsonous acid (MMA^III^), a highly genotoxic and cytotoxic intermediate [18]. Following this, AS3MT and SAM can methylate MMA^III^ to form dimethylarsinic acid (DMA^V^), a third intermediate. DMA is the least toxic inorganic arsenic intermediate and is readily excreted in the urine [18]. The final step is a reduction of DMA^V^ to DMA^III^; however, due to the high reactivity of DMA^III^, oxidation back to its earlier form DMA^V^ can occur rapidly [18].

Glutathione (GSH), a common antioxidant, can bind to arsenic and other toxins in the blood and catalyze reduction reactions, including those presented above [18,22]. In 2005, Hayakawa et al., identified that arsenic-glutathione (As-GSH) complexes are a necessary substrate for AS3MT activity and can catalyze the reactions described by Challenger [23]. As a result, more recent studies have proposed an altered pathway for arsenic metabolism, whereby As-GSH complexes activate AS3MT and consequently break down inorganic arsenic into similar MMA^III^ and DMA^V^ compounds [5,18,23]. More research is needed to better elucidate the true pathway of inorganic arsenic metabolism to facilitate the development of tangible interventions to promote successful arsenic metabolism. 

Exposure to arsenic can stimulate epigenetic disruption on various cellular processes, which can cause cancer. These disruption processes have been well characterized in previous literature and will be succinctly reviewed here. Briefly, inorganic arsenite can inhibit DNA mismatch repair, promoting genomic instability; activate pathways associated with unregulated cell proliferation, stimulating the transition of epithelial to mesenchymal cells; stimulate inflammation and angiogenesis through activation of NFkB and VEGF; increase cellular tyrosine phosphorylation, leading to aberrant cell signaling and the accumulation of reactive oxidative species contributing to cell death [10,19,24,25]. Previous work has also found that exposure to inorganic arsenic can result in chromosomal abnormalities, the stimulation of sister chromatid exchange, and the silencing of DNA methyltransferases which can inhibit the cell repair cycle and additionally interfere with important tumor suppressor genes, such as P16 [25]. Further, the arsenic metabolic pathway uses methyl stores from SAM, and can contribute to global DNA hypomethylation [25].

The methylated intermediates of arsenic can also increase the risk of cancer. The fraction of MMA to DMA detected in the urine acts as an indicator for increased susceptibility for arsenic related cancers [5,18,26,27]. Previous scholarship has established that the methylation status of arsenic’s intermediates is a defining feature of the element’s carcinogenicity. It has also been determined that high levels of toxic intermediate species (such as MMA) in the urine signal incomplete detoxification of the element, suggesting circulation and accumulation of highly genotoxic and cytotoxic intermediates in the blood and surrounding tissues (including the heart, liver, lungs and kidneys) [2,26,28]. Therefore, high levels of toxic arsenic species in the urine may promote many of the carcinogenic pathways presented above. 

## 3. Biomarkers of Arsenic Status

Arsenic can be detected in various biological samples and quantified using several biomarkers as a way to asses human exposure to the toxin. Scalp hair, nail samples, blood and urine are the most commonly utilized biomarkers of arsenic exposure and are indicative of systemic absorption of the toxin. Each of these biomarkers have been well characterized in previous literature, and an overview of each biomarker, common procedures for arsenic quantification, measured outcomes and their meanings are summarized in Table 1. Biomarkers measure cumulative exposure to a toxin; however, they only reflect levels of exposure for a specific timeframe, a critical aspect that must be accounted for when interpreting any study results.

Arsenic absorbed by the body binds to sulfhydryl groups, and can accumulate in keratin-dense tissue such as scalp hair and nails [29,30]. Arsenic measured in these slow growing tissues reflect long-term levels of exposure to the toxin, and can be indicative of exposure from 3–6 months prior [30,31,32,33,34]. For this reason, hair and nail biomarkers are commonly used to quantify levels of exposure in studies where the population of interest has been exposed to high levels of arsenic for an extended period of time (specifically when the primary route of exposure is from drinking water) [30]. Additionally, arsenic measured in scalp hair and nails only quantifies levels of iAs exposure. Previous animal studies have found that organic arsenic species (prominent in fish and seafood) do not accumulate in hair and nails, thus providing researchers a more accurate estimation of exposure to the carcinogenic forms of arsenic [30]. However, these biomarkers fail to account for the differentiation between internal exposure to arsenic (reflective of the burden on internal organs and systems), which is of interest, and external exposure (such as bathing in contaminated water) [35,36].

In contrast, arsenic measured in the blood and the urine reflects more recent exposure to the toxin, due to the short half-life of arsenic in these mediums following absorption (approximately 2–6 h in the blood, and 4 days in the urine) [33,35,36]. Even though both biomarkers reflect a similar timeframe of exposure, blood and urinary arsenic levels are suggestive of different biological burdens. Arsenic concentrations measured in blood plasma are reflective of internal exposure to the toxin, and directly describe the burden of exposure on specific organs and tissues [37]. Blood arsenic is commonly used to assess recent, high levels of exposure; however, it can also be used in dose-response studies to assess chronic levels of exposure from a wide-array of sources, including water, diet and occupation-specific exposure [36]. Recent work has also established that levels of iAs in drinking water are significantly associated with plasma arsenic, confirming its ability to reliably be used to quantify iAs exposure [38]. However, several limitations exist to solely measuring exposure through blood arsenic. Unlike arsenic measured in hair and nail samples, total blood arsenic levels represent comprehensive exposure to the toxin, including levels of non-toxic organic arsenic species. Therefore, it is necessary to accurately quantify levels of inorganic arsenic, and its metabolites, using speciation techniques when using blood plasma or provide study participants with clear instructions to abstain from consuming foods with high levels of oAs (e.g., fish and seafood) prior to blood draw [30]. Moreover, blood matrices are more challenging to work with and require invasive collection techniques, which may not be feasible for large cohort studies [30,36].

Urinary arsenic biomarkers are used to quantify the levels of arsenic excretion but cannot be used to describe the burden of arsenic exposure on specific organs and tissues [37]. Despite this limitation, urinary arsenic biomarkers are widely utilized, and often regarded as the gold-standard for assessing arsenic exposure, as they reliably and non-invasively quantify recent exposure to the toxin [34,39,40]. Since the urine is the primary method of excretion for all arsenic species absorbed by the body, and urinary arsenic concentrations can be speciated to quantify levels of relevant arsenic metabolites, it is commonly utilized to assess exposure in large cohort and population studies [36]. An important limitation of using this biomarker is revealed when discussing the type of sample collection that is used (ex. 24-h collection vs. spot collection [including first-morning void]). 24-h collection, which is considered the preferred method to assess biomarkers in the urine, is often not feasible for large cohort studies because of logistic and cost-prohibitive barriers [36]. For this reason, spot collection samples are commonly collected, though results can be biased because of variations in sample dilutions [36,41]. It is therefore critically important to adjust for an individual’s hydration status when conducting these analyses, which is regularly accomplished by normalizing urinary arsenic levels with urinary creatinine. However, previous work in the field has established an association between urinary creatinine and levels of urinary arsenic metabolites [36,41]. To address this, literature suggests the best practice is to include urinary creatinine as an independent variable in regression analysis; though, uptake of this methodology in current literature has been slow [41]. Finally, similar to blood arsenic, total arsenic levels measured in the urine are reflective of comprehensive exposure to both toxic and non-toxic arsenic compounds. Therefore, speciation of urinary arsenic is vital in order to accurately assess and quantify exposure to inorganic arsenic.

**Table 1:** Overview of biomarkers of arsenic status.

Exposure Measurement	Time Frame of Exposure	Type of Arsenic Measured	Method of Measurement	Toxic Dose ^1^
Scalp Hair	6–12 months prior	iAs	Options: Atomic Absorption Spectrometry Neutron Activation Analysis	1.0 < 3.0 mg/kg
Toenail	6–12 months prior	iAs	Options: Inductively Coupled Plasma Mass Spectrometry (ICP-MS) Neutron Activation Analysis	>0.5 µg/g
Blood Arsenic	2–6 h prior	Total arsenic Speciated arsenic intermediate levels (iAs^III^, iAs^V^, MMA^III^, MMA^V^, DMA^III^, DMA^V^, Ab, Ac and other oAs species)	High Performance Liquid Chromatography (HPLC) ICP-MS Anion Exchange	>130 nmol/L
Urinary Arsenic	4 days prior	Total arsenic Speciated arsenic intermediate levels (iAs^III^, iAs^V^, MMA^III^, MMA^V^, DMA^III^, DMA^V^, Ab, Ac and other oAs species)	HPLC ICP-MS Anion Exchange	>100 ug/L (24- h) [41] >50 μg/L (spot)

iAs: Inorganic arsenic, MMA: monomethylarsonic acid, DMA: dimethylarsinic acid, Ab: Arsenobetaine, Ac: Arsenocholine. ^1^ Indicates acute poisoning.

## 4. Arsenic and Breast Cancer: Ecologic and Prevalence Studies

There have been a limited number of ecologic and prevalence studies investigating the relationship between arsenic exposure and breast cancer risk. Table 2 summarizes the key characteristics and findings of the five studies that have estimated population-level exposure to arsenic using recorded levels from soil, water and air. Despite limitations associated with assigning proxy levels of exposure to large groups, potential regional variation in measurement and lack of data available on potential confounders, the findings from these studies provide important preliminary evidence suggesting a potential relationship between level of arsenic exposure and breast cancer risk [42,43].

In the first study, Hinwood et al. investigated the association between inorganic arsenic exposure and cancer incidence in 22 regions of Victoria, Australia where elevated levels of inorganic arsenic were reported in soil, water or both environmental media [44]. Using the Victoria Cancer Registry and Victorian cancer rates, the authors reported increased standard incidence ratios (SIR) for breast cancer across all regions (SIR = 1.10, 95% confidence interval [CI] 1.03–1.18); however, no association was reported after stratifying by similar regional characteristics (elevated soil iAs/elevated water iAs/elevated in both environmental media) likely due to low power [44]. In 2008, Baastrup et al., similarly investigated the relationship between lifetime exposure to arsenic via drinking water, and cancer risk for two regions in Denmark (Aarhus and Copenhagen) using time-weighted average exposure and cumulative exposure metrics [45]. After stratification by region, the authors reported a marginally significant association between increased time-weighted arsenic exposure and elevated risk of breast cancer in Aarhus (Incidence Rate Ratio [IRR] = 1.06, 95%CI 1.01–1.11, *p* = 0.002) [45]. A comparable association was observed across the United States Surveillance, Epidemiology and End Results program, where a study investigating the density of airborne arsenic emissions and breast cancer also reported a significant, positive association, after adjusting for relevant confounders (Regression coefficient (*B*) = 5.21, 95%CI 1.72–8.70, *p* = 0.004) [46].

Given the high levels of exposure associated with arsenic-laden drinking water, a study conducted by Aballay et al., revealed unexpected results when examining the relationship between levels of arsenic detected in well-water, and overall cancer burden in Cordoba, Argentina [47]. The authors reported no significant association between the level of arsenic in well-water, the primary form of drinking water for this area, and risk of breast cancer (IRR = 1.09, 95%CI 0.74–1.60) [47]. A similar result was reported in a British study examining cancer burden attributable to occupational exposures to various Type 1 and 2A IARC carcinogens, including airborne arsenic [48]. The authors did not report an attributable fraction (AF) for the association between occupational arsenic exposure and breast cancer but did estimate over 50% of all breast cancer would be attributable to shift-work exposure (AF = 1969/3616) [48].

## 5. Arsenic and Breast Cancer: Case-Control Studies

There are 10 case-control studies that have evaluated the relationship between arsenic exposure and breast cancer. The key features of these studies are summarized in Table 3. While the most common biomarkers used to assess arsenic exposure are hair or urinary arsenic concentrations, there have been a few studies that quantified arsenic levels directly in breast tissue. 

Prior to studies examining the explicit relationship between arsenic exposure and breast cancer, a series of case-control studies were conducted to determine if arsenic levels were elevated for individuals with breast cancer, compared to the general population. Six early case-control studies, with very small sample sizes, found mixed associations between levels of arsenic measured in hair or breast tissue of women with breast cancer, compared to healthy controls [49,50,51,52,53,54]. This work, though indirect, formed the biological rational for further investigation into the relationship between systemic exposure to arsenic, and in the development of breast cancer.

Since 2014, four case-control studies have been conducted using a unique cohort of women from five states in northern Mexico. The regions selected for recruitment into this cohort had previously been identified as areas with endemically high levels of arsenic in the population’s drinking water supply (surpassing the WHO limit of 10 ug/L); however, prior to commencement of the studies, these regions had taken drastic steps to reduce arsenic concentrations in drinking water to comply with the WHO standard. These important studies not only investigated the association between arsenic exposure and subsequent odd of breast cancer, but also identified important genetic differences in cases and controls that may account for this elevated risk. In the first study, Lopez-Carrillo et al., examined an individual’s ability to methylate inorganic arsenic, and their subsequent odds of breast cancer [55]. Through an analysis of speciated urinary arsenic metabolites in 1016 cases, and 1023 controls, the authors reported that arsenic exposure was associated with a significant, two-fold higher odds of developing breast cancer (Odds Ratio [OR] = 2.62, 95%CI 1.89–3.66), and established a precedent that the ability to methylate arsenic subspecies plays an integral role in this estimation [55]. This study was the first to report that women with a higher primary methylation index (PMI), the elevated capacity to methylate inorganic arsenic compounds into MMA, and reduced capacity to perform the second methylation step (from MMA to DMA), were at a higher odds of developing breast cancer (OR = 1.90, 95%CI 1.39–2.59) [5]. In 2016, this work was validated in a nested case-control analysis of urinary arsenic metabolites from 197 cases and 220 controls within the same cohort [56]. The authors reported that women within the highest tertile of primary methylation indexes (largest discrepancies between successful methylation of the two steps) were at an increased odds of developing breast cancer, compared to the referent, after adjusting for genetic polymorphisms (OR_PMI T3 vs.T1_ = 3.51, 95%CI 1.96–6.28) [56].

Building on this, Gamboa-Loira et al., studied whether the association between arsenic exposure and breast cancer varied by single nucleotide polymorphisms in the genes related to AS3MT and methionine synthase enzymes, both of which are essential for adequate arsenic metabolism [57]. The findings from this work revealed that an A > G polymorphism on MTR c.2756, a gene required for methionine synthase, significantly increased an individual’s capacity to methylate MMA to DMA, thereby, aiding the successful elimination of iAs from the body [57]. This polymorphism confers a protective effect on breast cancer for those carrying the homozygous (GG) or heterozygous (AG) variant [58]. Most recently, Lopez-Carrillo et al., investigated the impact of inorganic arsenic methylation capacity, on breast cancer by hormone receptor subtype [59]. The authors reported higher ratios of MMA detected in the urine, compared to unmethylated inorganic arsenic compounds, in some breast cancer subtypes. Specifically, this altered ratio was found to increase the odds of developing hormone receptor positive (estrogen receptor [ER] positive, or progesterone receptor [PR] positive, and human epidermal growth factor receptor 2 negative) cancers (OR = 2.03; 95%CI 1.33–3.10), and triple negative cancers (OR = 4.05;95%CI 1.63–10.04); although the confidence intervals were large [59]. This work is the first to suggest that the inorganic arsenic metabolite MMA may be most related to breast cancer carcinogenesis; however, further research in this area is needed to better characterize this potential mechanism of action and validate this association [59].

## 6. Arsenic and Breast Cancer: Prospective Studies

There are seven prospective studies of arsenic status and breast cancer, which have been summarized in Table 4. Garland et al., was the first to explore this relationship in a nested case-control study of 892 individuals within the Nurses’ Health Study cohort (NHS1), quantifying arsenic exposure in toenail samples [60]. After five years of follow up, the authors reported no association between arsenic status and breast cancer risk (OR_Q5 vs. Q1_ = 1.12, 95%CI 0.66–1.91, *p*-trend = 0.78) [60]. To date, there have been two studies that have used food frequency questionnaires to quantify arsenic exposure from diet alone. The first study, using an arsenic-specific 75-item food frequency questionnaire to assess exposure, found no association between level of arsenic exposure and breast cancer risk in a Japanese population (Hazard Ratio [HR] = 1.06, 95%CI 0.8–1.41, *p* = 0.35) [61]. In a similar study, where arsenic exposure was assessed using rice consumption as a proxy, Zhang et al., evaluated the impact of long-term rice consumption on cancer risk within the Nurses’ Health Study and the Health Professionals Follow-up study cohorts [62]. After 26 years of follow-up, 31,655 incident cancers were detected in the cohort, of which 8115 were breast cancers [62]. The authors reported no association between total rice consumption and breast cancer risk (Relative Risk (RR) = 0.90, 95%CI 0.70–1.16, *p* = 0.48) [62]. Most recently, Marciniak et al., examined this relationship in a cohort of 1703 Polish women [63]. Unlike other studies, Marciniak et al., reported a highly significant, 13-fold increased risk of breast cancer for women in the highest quartile of circulating blood arsenic levels, compared to the referent group (HR_Q4 vs. Q1_ =13.2, 95%CI 4.0–43.0, *p*-trend < 0.0001) [63].

There has also been an increasing number of prospective studies investigating the health implications of airborne arsenic exposure, using census tract data from the United States. These studies have largely reported null associations between airborne arsenic exposure and breast cancer risk. In the California Teacher’s cohort, Liu et al., reported no association between increasing levels of airborne arsenic exposure in residential areas and breast cancer risk (HR_Q5 vs. Q1_ = 1.1, 95%CI 0.9–1.2) [64]. However, a subgroup analysis of 245 ER and PR negative cancers revealed an increased risk of developed these breast cancer subtypes as levels of airborne arsenic increased (HR_Q5 vs. Q1_ = 1.7, 95%CI 1.1–2.5) [64]. When this relationship was investigated in a similarly designed study of 50,844 women from the Sister Study cohort, White et al., also reported no association between airborne arsenic exposure and breast cancer risk, overall, and by menopausal status (HR _Q5 vs. Q1_ = 1.0, 95%CI 0.9–1.2, *p*-trend = 0.6) [65]. A related finding was also reported in the Chicago Breast Cancer cohort, where no association was reported between level of arsenic exposure measured 3–6 years prior to breast cancer diagnosis, and odds of having an ER or PR negative breast cancer (OR_ER/PR negative breast cancers_ = 0.8; 95%CI 0.5–1.5) [66].

## 7. Clinical Implications and Future Directions

As evidenced in this review, human exposure to arsenic is inevitable. The widespread nature of arsenic contamination, coupled with its chronic existence within commonly consumed foods and water supplies, underscores its prominence as a global public health concern. Despite the prevalence of human interaction with this recognized carcinogen, this review revealed that the relationship between arsenic exposure and breast cancer risk remains unclear. As exposures to environmental toxins, including arsenic, represent a large and modifiable risk factor for breast cancer, there is an evident need for more prospective studies, which use appropriate biomarker to assess cumulative levels of exposure, to be performed. Research in this area is essential for developing instrumental policy, and crucial public health interventions, that are needed to reduce breast cancer incidence arising from arsenic exposure.

Biomarker studies are routinely performed to assess an individuals’ cumulative exposure to arsenic, as levels detected in these samples encompasses daily exposure from a multitude of sources, including the air, diet and water. Despite this, approximately half of the prospective studies included in this review exclusively evaluated arsenic exposure from one source (be it in the air or from the diet), failing to account for the critically important contribution that other sources of arsenic may have on total exposure levels. This limitation is also of concern for ecologic studies, which do not directly assess individual’s arsenic exposure, or account for variations in exposure level when calculating risk estimates. Previously, Kurzius-Spencer et al., estimated that when drinking water meets provisional standards, approximately 30% of daily inorganic arsenic exposure can be attributed to diet alone [67]. This underscores the need to consider all sources of inorganic arsenic exposure when determining the impact of this carcinogen on breast cancer risk, and the importance of correctly characterizing the burden of exposure. Future prospective studies should objectively assess cumulative arsenic exposure through use of a biomarker that can be speciated, such as urine or blood, to accurately assess comprehensive exposure to arsenic, and better elucidate the relationship between arsenic exposure and breast cancer risk.

A substantial number of case-control studies included in this review examined risk of breast cancer in geographic regions with endemically, or historically, high levels of arsenic. Though exposure levels for these populations are much higher, studies investigating risk in these regions do not appropriately reflect the global level of arsenic exposure. Although countries with historically high levels of arsenic within their drinking water have taken steps to comply with the WHO standard, these recent improvements do not negate the long-lasting implications that high levels of arsenic exposure can have during critical windows of breast cancer susceptibility (prenatal, puberty, pregnancy and menopausal transition) [68]. A higher level of exposure to endocrine disrupting hazards, such as arsenic, during these critical windows could disproportionally increase breast cancer risk for these populations, and current study efforts may not be reflective of the true association between arsenic exposure and breast cancer risk. This exposes two prominent questions that require further clarification from future studies. The first seeks to assess the true impact that chronic, low-dose exposure to arsenic has on cancer risk. The second question asks how exposure to high levels of arsenic, during critical periods of development, impacts subsequent breast cancer risk within populations that have recently reduced arsenic concentrations in drinking water.

Though it is well known that the dose makes the poison, the inconclusive association between arsenic exposure and breast cancer calls into question the dose of arsenic necessary to deem it carcinogenic. Whether it be exposure to large or small quantities of arsenic compounds, it remains unknown if arsenic exposure is indeed associated with breast cancer risk. If arsenic exposure does increase breast cancer risk, the omnipresent nature of this compound represents a large risk factor, to which exposure can be drastically reduced through effective, national and international, public health policy.

## Figures and Tables

**Table 2:** Summary of ecologic studies investigating the relationship between arsenic exposure and breast cancer risk.

Author and Study Year	Location of Study	Exposure Measurement	Sample Size	Registry	Outcome	*p*-Value	Association
Hinwood et al. 1999 [44]	Australia	Arsenic in soil + surface water	22 areas	Victorian Cancer Registry data & Victorian cancer rates	Standardized Incident Ratio (SIR) (95%CI) SIR= 1.10 (1.03–1.18)	N/S	Positive
Baastrup et al. 2008 [45]	Denmark (Aarhus and Copenhagen)	Arsenic in water	29,502	Geological Survey of Denmark&Danish Cancer Registry	Incident Rate Ratio (IRR) for time weighted exposure (95%CI) IRR_ARH_= 1.06 (1.01–1.11)	0.02	Positive
Rushton et al. 2010 [46]	Britain	Occupation (CAREX)	339,156 total cancer registrations (2004)	Office for National Statistics; General Register Office (Scotland); Cancer Statistics, Registrations, Series MB1 for England; The Scottish Cancer Registry; The Welsh Cancer Intelligence and Surveillance Unit	Attributable Fractions N/S	N/S	Null
Aballay et al. 2011 [47]	Cordoba, Argentina	Arsenic in water	123 rural regions	Córdoba Cancer Registry & 2004 National Well Monitoring Reports	Incident Risk Ratio (95% CI) IRR = 1.09 (0.74–1.6)	N/S	Null
Vu et al. 2019 [48]	USA	Arsenic in air	200 counties	Surveillance, Epidemiology and End Results (SEER) & 2008 National Emissions Inventory (NEI)	Regression coefficient for change in annual incidence of breast cancer and emission density of arsenic B_All BC_ = 5.21 (1.72, 8.70) B_ER + BC_ = 4.15 (0.87, 7.43)	0.004 0.014	Positive

N/S: not stated, BC: breast cancer, ER+: estrogen receptor positive.

**Table 3:** Summary of case-control studies investigating the relationship between arsenic exposure and breast cancer occurrence.

Author and Study Year	Exposure Measurement	Location of Study	Sample Size	Referent Group	Outcome	*p*-Value	Association
Garg et al. 1996 [49]	Arsenic in breast tissue	India	30 cases/30 controls	Case vs. control	Change in mean value of arsenic ^1^ 7.8%	N/S	Positive
Joo et al. 2009 [50]	Hair	South Korea	40 cases/144 controls	Case vs. Control	Mean ± Standard Error Cases: 0.09 ± 0.006 Controls: 0.06 ± 0.003	<0.001	Positive
Alatise et al. 2010 [51]	3 exposures: Whole blood Scalp hair Breast biopsy	Nigeria	12 cases/ 12 controls	Case vs. Control	Mean Concentrations of arsenic by biomarker Whole Blood Controls – 6.8 µg/L (4.0–12) Cases- 7.6 µg/L (3.4–16) Scalp Hair Controls – 0.09 (0.02–0.18) Cases – 0.08 (0.004–0.18) Breast Biopsy Median- 0.077 mcg/g (0.032–0.11)	Student’s *t*-Test 0.11 0.28	Null
Benderli Cihan et al. 2011 [52]	Hair	Turkey	52 cases/ 52 controls	Case vs. Control	Mean ± (Standard Deviation) Cases: 1.522 ug/g (1.980) Controls: 0.239 ug/g (0.220)	<0.05	Positive
Blaurock-Busch et al. 2013 [53]	Hair	India	15 cases/50 controls	Case vs. Control	Mean concentration difference between healthy control and cases 0.11 ug/g	N/S	Null
Lopez-Carrillo et al. 2014 [54]	Urinary arsenic	Mexico	1016 cases/1028 controls	Q5 vs. Q1	Odds Ratio (OR) (95%CI) OR_MMA_ = 2.63 (1.89–3.66) OR_PMI_ = 1.90 (1.39–2.59)	*p* for trend < 0.001	Positive
Wadhwa et al. 2015 [55]	Hair	Pakistan	47 cases/94 controls	Case vs. Control	Standard Mean Difference 2.94 (2.77–3.12)	<0.05	Positive
Pineda-Belmontes et al. 2016 [56]	Urinary arsenic	Mexico	197 cases/220 controls	T3 vs. T1	Odds Ratio (95%CI) OR_MMA_ = 3.57 (1.99–6.38) OR_PMI_ = 3.51 (1.96–6.28)	N/S	Positive
Gamboa-Loira et al. 2017 [57]	Urinary arsenic	Mexico	1016 cases/ 1028 controls	MTR AA vs. MTR AG + GG	Odds Ratio (95%CI) OR_BCwith%DMAamong*MTR*AA_ = 0.86 (0.54–1.38)	*p* for interaction = 0.002	Positive
Lopez-Carrillo et al. 2020 [58]	Urinary arsenic	Northern Mexico	499 cases/499 controls	Q5 vs. Q1	Odds Ratio (95%CI) HR+ BC OR_MMA/iAs continuous_ = 2.03 (1.33–3.10) TN BC OR _MMA/iAs continuous_ = 4.05 (1.63–10.04)	N/S	Positive

N/S: not stated, T: tertile, MMA: monomethylarsonic acid, DMA: dimethylarsinic acid, iAs: inorganic arsenic, PMI: primary methylation index, BC: breast cancer, HR + BC: hormone receptor positive breast cancer (estrogen receptor positive and/or progesterone receptor positive, and human epidermal growth factor receptor 2 negative), TN: triple negative breast cancer (estrogen receptor negative and/or progesterone receptor negative, and human epidermal growth factor receptor 2 negative). ^1^ Change- Ratio of change in mean values and mean concentration in normal tissue × 100.

**Table 4:** Summary of prospective studies investigating the relationship between arsenic exposure and breast cancer risk.

Author and Study Year	Exposure Measurement	Location of Study	Sample Size	Follow Up (years)	Referent Group	Outcome	*p*-Value	Association
Garland et al. 1996 [59]	Toenail	USA	433 BC cases/459 controls	4	Q5 vs. Q1	Multivariate Odds Ratio (95% CI) OR = 1.12 (0.66–1.91)	*p* for trend = 0.78	Null
Sawada et al. 2013 [60]	75- item arsenic specific food frequency questionnaire	Japan	7002 incident cancers/ 90,378 total individuals	11	Q4 vs. Q1	Multivariate Hazards Ratio (HR) (95% CI) HR = 1.06 (0.8–1.41)	0.35	Null
Liu et al. 2016 [61]	Airborne arsenic	USA	5361 BC cases/112,379 total individuals	15 ^1^	Q5 vs. Q1	Cox proportional hazards ratio (95%CI) HR = 1.1 (0.9–1.2) HR _ER/Pr – Subtype_ = 1.7 (1.1–2.5)	N/S	Null Positive
Zhang et al. 2016 [62]	Rice consumption as a proxy for arsenic exposure	USA	31,655 incident cancers/206,249 total individuals	26	>5 servings of rice/week vs. <1 serving of rice/week	Relative Risk Ratio (RR) (95%CI) RR = 0.90 (0.70–1.16)	0.48	Null
Kresovich et al. 2019 [63]	Airborne arsenic	USA	672	3–6	Q5 vs. Q1	Odds Ratio (95% CI) OR_ER/PR – Subtype_ = 0.8 (0.5–1.5)	0.89	Null
Marciniak et al. 2019 [64]	Total blood arsenic	Poland	1702	4.5	Q4 vs. Q1	Cox proportional hazards ratio (95%CI) HR = 13.2 (4.02–43.0)	*p* for trend < 0.0001	Positive
White et al. 2019 [65]	Airborne arsenic	USA	50,884	7.4	Q5 vs. Q1	Cox proportional hazards ratio (95%CI) HR_Overall BC_ = 1.0 (0.9–1.2)	0.6	Null

N/S: not stated, ER-: Estrogen Receptor negative, PR-: Progesterone receptor negative, BC: breast cancer. ^1^ Study conducted from 1995–2010, however, average follow-up time was not explicitly stated.
